# *BJUI Compass* 2021 year‐end review

**DOI:** 10.1002/bco2.122

**Published:** 2021-11-17

**Authors:** John W. Davis

**Affiliations:** ^1^ BJUI Compass

Greetings to our *BJUI Compass* audience. We have already reached the end of our 2nd year of existence as the first new journal to add to the BJUI family. We are a fully digital, open access journal that is fully peer reviewed to the same standards as the BJUI. We accept all subspecialties within clinical urology and group our papers by our four key “directional” compass themes of review articles, clinical utility, academic progress, and surgical innovation. Our board consists of an editor, two Associate Editors, and 12 Consulting Editors. All accepted papers go online quickly after formatting and are then assigned an issue. Our social media editor is Zainal Adwin who tweets summaries of our papers, and we have a YouTube channel for video supplements any authors want to post.

Papers submitted to *BJUI Compass* come by many routes: Approximately 35% are transferred from the *BJUI* as suitable content for *BJUI Compass* and the remaining from direct submissions. An increasing number of author groups are finding they have article charges covered by their institution's open access agreements with Wiley and the list is posted via links from our website. We strive to cover all aspects of urology and last year saw an equal 50:50 ratio of submissions from urologic oncology to functional/other areas of urologic practice. Of course, our initial issue in March 2020 coincided with the COVID‐19 pandemic, and a number of articles are now available on the effect of the pandemic on urologic practice. Our initial issues had at least four articles/issue, and this past year, we are at around eight/issue. Overall, we had 71 submissions in 2020 and this year on pace for 90.

In conclusion, it has been a satisfying growth trend for *BJUI Compass*. We look forward to furthering the science in our field in 2022. In the meantime, Figure 1 shows a glimmer of hope regarding in‐person events post pandemic.

**FIGURE 1:**
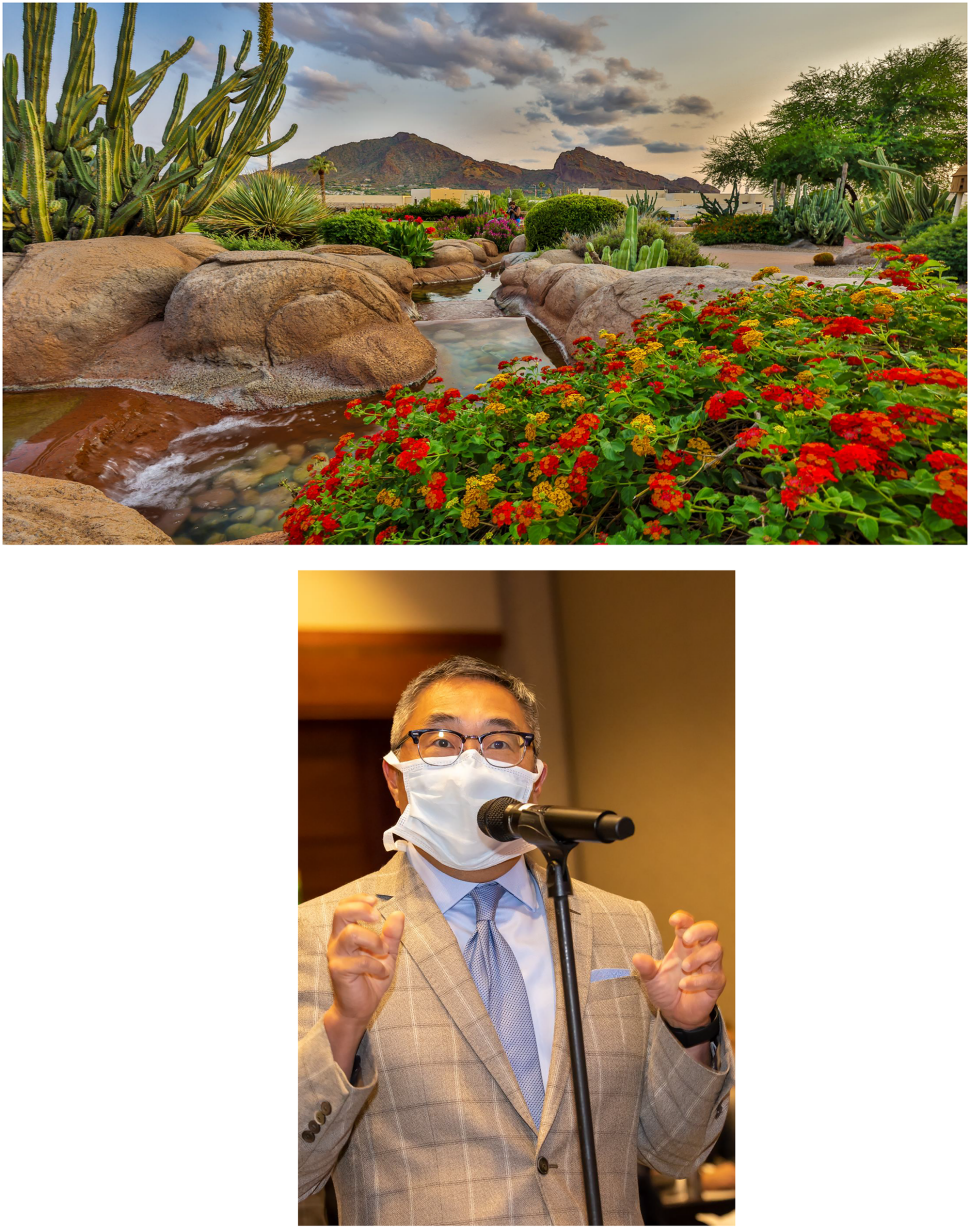
Are meetings back for 2022? In the United States in late 2021, a handful of small/regional meetings hosted in‐person meetings with multiple Covid‐19 protocols, and virtual options for speakers who could not travel. (a) A return to the peaceful resort meetings such as the Camelback Inn in Arizona, which hosted the 100th meeting of the South Central Section of the AUA. (b) The new look for pandemic/endemic meetings? Discussion time at the 100th South Central AUA after a panel discussion on active surveillance for prostate cancer

For the November 2021 *BJUI Compass*, we have nine articles …

- *To the Journals*… The narrative review by Thakare et al.^1^ encompassed 1970–2020 articles on orthotopic bladder substitution for patients undergoing cystectomy. The paper highlights the full pathway of preoperative planning, intraoperative results, and postoperative management. For trainees and young faculty, the authors supplement their narrative with a set of illustrations for different pouches, and several tables that can serve as checklists for reviewing operative planning, and counselling on complications.
- *To the Clinic*… Prostate cancer diagnostic management is increasingly shifting towards MRI as part of all initial biopsy decisions and, potentially, technique. Meanwhile, secondary biomarkers are commercially available that aim to improve the specificity of PSA screening. Carbunaru et al.^2^ looked at a biopsy‐naïve cohort that had both mpMRI and prostate health index—an emerging concept of trying to combine biomarkers with imaging (and treating imaging like a part of a biomarker performance metric). Their key finding was that use of PHI with PiRADS 3 lesions avoided 55% of unnecessary biopsies. Of interest, their cohort had >40% African Americans and several observations noted and discussed. For example, African American race was a predictor of GG2‐5 prostate cancer, but did not modify the effect of PHI, PSA density, or PiRADS scores.
  Furthering our progress in prostate cancer diagnostics, Tay et al.^3^ performed a deep dive into prostate MRI metrics at an Australian referral center. They found a negative predictive value of 96% at PiRADS 2 or lower. Of clinically significant cancers, 94% were PiRADS 3 or above. Interestingly, they were not able to show improved diagnostic performance when adding MRI to the ERSPC risk calculator.
  Stone et al.^4^ looked at the transperineal prostate biopsy method as a way to evaluate men with an abnormal MRI who might consider focal therapy. In this series, the transperineal was more of a saturation method and was positive in 53% for clinically significant disease. Overall, the authors grouped the patients by biopsy and MRI and demonstrate concordance analyses. They argue common points about how to supplement MRI with biopsy data and determine if focal therapy is feasible, and which extent of ablation zone would be required.
  Maddan et al.^5^ performed a timely article on nurse‐led one stop hematuria clinics in the United Kingdom with a sample size of 2714 patients—including nurse performed flexible cystoscopy and ultrasound. Their data separate malignancy findings by gender, visible versus non‐visible hematuria, and site of malignancy in the genitourinary tract. Positive cases were referred for rigid cystoscopy, TURBT, and CT urography. Overall, 59% were discharged/reassured with no pathologic findings. In part, this work flow derives from a country‐specific mandate to see these patients within a 2‐week referral window. This topic is rich with follow‐up questions, including the accuracy of patients cleared from their hematuria work‐ups and how training/supervision is conducted.

- *To the Drawing Board*… Renal cell carcinoma remains a common cancer that lacks formal/intended screening methods and rather relies upon incidental findings from imaging. There remain multiple challenges to optimize and improve therapy for advanced disease. Gundert et al.^6^ performed an immunohistochemistry level study of a class of biomarkers called m^6^A writers, which are a class of post‐transcriptional RNA modifiers, and it is known that dysregulation at these sites contributes to cancer and benign conditions (obesity and diabetes). The authors correlated expression levels with histologic grades and found patterns consistent with a tumor suppressor activity.
  Pattenden et al.^7^ performed an interesting and emerging space study of erectile dysfunction with a focus on the quality/readability of patient online information. Imagine having Google as your data collection base. Of interest, they graded information using several validated measures. Overall, the quality of online information was poor, but they noted that sites that listed specific certifications or benchmarks did better. If you are asked to help your hospital with patient level online information, this paper may provide effective guidance.
  Touching upon the topics of COVID‐19 and prostate cancer treatment, Barrett et al.^8^ looked at a pharmacy level of data collection for androgen deprivation treatment plans and found a concerning downward trend in prescriptions. This is basically a way to study how pandemic lockdowns may have unintended health consequences.

- *To the Future*… We previously touched upon the transperineal biopsy as a method of selecting patients for focal therapy. In the article by Hogan et al.,^9^ we feature the technique of this biopsy along with the anesthesia strategy. In this prospective cohort study, the authors show tolerability for local anesthesia only—in addition to shorter procedure time, recovery time, and total time in facility.

## References

[1] Thakare N , Lamb BW , Biers S . Orthotopic bladder substitution: surgical aspects and optimization of outcomes. BJUI Compass. 2021;00:1–11. 10.1111/bco2.84 PMC8988640 35474698

[2] Carbunaru S , Stinson J , Babajide R , Hollowell CM , Yang X , Sekosan M , et al. Performance of prostate health index and PSA density in a diverse biopsy‐naïve cohort with mpMRI for detecting significant prostate cancer. BJUI Compass. 2021;00:1–7. 10.1111/bco2.91 PMC8988695 35474697

[3] Tay JYI , Chow K , Gavin DJ , Mertens E , Howard N , Thomas B , et al. The utility of magnetic resonance imaging in prostate cancer diagnosis in the Australian setting. BJUI Compass. 2021;00:1–8. 10.1111/bco2.99 PMC8988779 35474704

[4] Madaan A , Kuusk T , Hamdoon M , Elliott A , Pearce D , Madaan S . Nurse‐led one stop hematuria clinic: outcomes from 2,714 patients. BJUI Compass. 2021;00:1–10. 10.1111/bco2.100 PMC8988527 35474702

[5] Stone N , Skouteris V , Chang S , Klimis A , Lucia MS . Transperineal prostate biopsy identifies locations of clinically significant prostate cancer in men considering focal therapy with PI‐RADS 3–5 regions of interest. BJUI Compass. 2021;1–7. 10.1002/bco2.111 PMC8988820 35474703

[6] Gundert L , Strick A , von Hagen F , Schmidt D , Klümper N , Tolkach Y , et al. Systematic expression analysis of m6A RNA methyltransferases in clear cell renal cell carcinoma. BJUI Compass. 2021;00:1–10. 10.1111/bco2.89 PMC8988738 35474700

[7] Pattenden TA , Raleigh RA , Pattenden ER , Thangasamy IA . Quality and readability of online patient information on treatment for erectile dysfunction. BJUI Compass. 2021;00:1–7. 10.1111/bco2.87 PMC8988690 35474701

[8] Barrett R , Barrett R , Dhar K , Birch B . Gonadorelins adherence in prostate cancer: a time‐series analysis of England's national prescriptions during the COVID‐19 pandemic (from Jan 2019 to Oct 2020). BJUI Compass. 2021;00:1–9. 10.1002/bco2.101 PMC8427122 34518826

[9] Hogan D , Kanagarajah A , Yao HH , Wetherell D , Dias B , Dundee P , et al. Local versus general anesthesia transperineal prostate biopsy: tolerability, cancer detection, and complications. BJUI Compass. 2021;1–8. 10.1002/bco2.106 PMC8988812 35474705

